# Supplementary material for: Modeling glioblastoma heterogeneity as a dynamic network of cell states
Source: Mol Syst Biol. 2021 Sep 16;17(9):e10105. doi: 10.15252/msb.202010105 (PMC8444284; doi:10.15252/msb.202010105)
Supplement: Supplementary file 6 — Source Data for Figure 5 [file MSB-17-e10105-s004.zip › Figure5A_sourcedata/GSEA_3017/nosigdb_stateB.GseaPreranked.1622795021725/RICHARDS_NOWAKOWSKI_SCIENCE_2017_OPC_UPREG.html]

Details for gene set RICHARDS\_NOWAKOWSKI\_SCIENCE\_2017\_OPC\_UPREG[GSEA]

|  || Dataset | state43017 |
| Phenotype | NoPhenotypeAvailable |
| Upregulated in class | na\_neg |
| GeneSet | RICHARDS\_NOWAKOWSKI\_SCIENCE\_2017\_OPC\_UPREG |
| Enrichment Score (ES) | -0.42963892 |
| Normalized Enrichment Score (NES) | -2.9293802 |
| Nominal p-value | 0.0 |
| FDR q-value | 0.0 |
| FWER p-Value | 0.0 |
Table: GSEA Results Summary

  

Fig 1: Enrichment plot: RICHARDS\_NOWAKOWSKI\_SCIENCE\_2017\_OPC\_UPREG      
 Profile of the Running ES Score & Positions of GeneSet Members on the Rank Ordered List

  

| PROBE | GENE SYMBOL | GENE\_TITLE | RANK IN GENE LIST | RANK METRIC SCORE | RUNNING ES | CORE ENRICHMENT || 1 | LRRN3 |  |  | 25 | 0.704 | 0.0217 | No |
| 2 | LYPD1 |  |  | 151 | 0.459 | -0.1135 | No |
| 3 | PDE1C |  |  | 166 | 0.445 | -0.0973 | No |
| 4 | NTM |  |  | 209 | 0.409 | -0.1225 | No |
| 5 | TENM1 |  |  | 291 | 0.354 | -0.2056 | No |
| 6 | DDAH1 |  |  | 300 | 0.346 | -0.1890 | No |
| 7 | PKP4 |  |  | 311 | 0.342 | -0.1755 | No |
| 8 | PDGFA |  |  | 328 | 0.335 | -0.1708 | No |
| 9 | GRIA4 |  |  | 358 | 0.322 | -0.1850 | No |
| 10 | ACTN4 |  |  | 376 | 0.316 | -0.1832 | No |
| 11 | SLC35F1 |  |  | 386 | 0.312 | -0.1707 | No |
| 12 | BLM |  |  | 408 | 0.305 | -0.1753 | No |
| 13 | NUP50 |  |  | 470 | 0.287 | -0.2362 | No |
| 14 | PLOD2 |  |  | 474 | 0.286 | -0.2175 | No |
| 15 | GNG12 |  |  | 587 | 0.267 | -0.3501 | No |
| 16 | KHDRBS3 |  |  | 599 | 0.263 | -0.3443 | No |
| 17 | NLGN4X |  |  | 641 | 0.257 | -0.3802 | No |
| 18 | MYBL1 |  |  | 678 | 0.251 | -0.4097 | Yes |
| 19 | SCD |  |  | 688 | -0.263 | -0.4011 | Yes |
| 20 | CHD7 |  |  | 706 | -0.284 | -0.4018 | Yes |
| 21 | MMP16 |  |  | 707 | -0.285 | -0.3791 | Yes |
| 22 | DSEL |  |  | 709 | -0.297 | -0.3569 | Yes |
| 23 | MYO10 |  |  | 710 | -0.305 | -0.3326 | Yes |
| 24 | EPN2 |  |  | 711 | -0.305 | -0.3083 | Yes |
| 25 | ZEB1 |  |  | 717 | -0.317 | -0.2899 | Yes |
| 26 | FGFBP3 |  |  | 721 | -0.334 | -0.2674 | Yes |
| 27 | OLIG1 |  |  | 729 | -0.370 | -0.2476 | Yes |
| 28 | HAS2 |  |  | 733 | -0.389 | -0.2207 | Yes |
| 29 | ATCAY |  |  | 738 | -0.417 | -0.1930 | Yes |
| 30 | DBI |  |  | 740 | -0.422 | -0.1608 | Yes |
| 31 | PDGFRA |  |  | 758 | -0.673 | -0.1306 | Yes |
| 32 | FABP5 |  |  | 759 | -1.641 | 0.0000 | Yes |
Table: GSEA details [plain text format]

  

Fig 2: RICHARDS\_NOWAKOWSKI\_SCIENCE\_2017\_OPC\_UPREG: Random ES distribution      
 Gene set null distribution of ES for **RICHARDS\_NOWAKOWSKI\_SCIENCE\_2017\_OPC\_UPREG**
